# Unicystic Ameloblastoma of Mandible: A Case Report

**DOI:** 10.31729/jnma.7566

**Published:** 2022-07-31

**Authors:** Sushil Rayamajhi, Sunita Shrestha, Samata Shakya, Sagar Bhandari, Anu Radha Twayana, Kopila Shahi

**Affiliations:** 1 Department of Medicine, Swacon International Hospital, Rudramati Marg, Kathmandu, Nepal; 2 Department of Medicine, All Nepal Hospital Pvt. Ltd, Samakhusi, Kathmandu, Nepal; 3 Department of Medicine, Bhaktapur Hospital, Dudhpati, Bhaktapur, Nepal; 4 ZH Sikder Women's Medical College and Hospital, Gulshan, Dhaka, Bangladesh

**Keywords:** *ameloblastoma*, *odontogenic cysts*, *odontogenic tumors*, *segmental mandibulectomy*

## Abstract

Ameloblastomas of jaws are benign odontogenic tumors of epithelial origin with four clinical variants: solid multicystic type, unicystic type, desmoplastic type, and extraosseous type. The incidence rate of ameloblastoma is 0.92 per million person-years. Unicystic ameloblastoma refers to those cystic lesions that show clinical and radiologic characteristics of an odontogenic cyst but shows a typical ameloblastomatous epithelium lining part of the cyst cavity, with or without luminal and/or mural tumor proliferation on histological examination. Here is a unique case of unicystic ameloblastoma involving the mandible in a 70-year-old patient. The case was managed by segmental mandibulectomy and flap repair. Unicystic ameloblastoma accounts for only 13% of all known cases in scientific literature. Considering the rarity of the lesion, the purpose of presenting this report on a clinical case is to emphasize the importance of radiological evaluation and histopathological examination for the diagnosis of ameloblastoma.

## INTRODUCTION

Ameloblastoma is a benign odontogenic tumour with an aggressive growth tendency and a high risk for malignant transformation and metastasis.^1^ The pooled incidence rate of ameloblastoma is 0.92 per million person-years.^2^ They account for 1% of all oral tumours.^3^ The World Health Organization classifies ameloblastomas into four clinical categories as solid multicystic, unicystic, desmoplastic, and extraosseous/peripheral.^4^ Unicystic ameloblastoma (UA) is a distinct kind of ameloblastoma characterized by slow growth and is relatively locally aggressive.^5^ UA is a rare variant of ameloblastoma frequently encountered in second or third decade. This report highlights the importance of radiological and pathological examination of any pathology in the jaw even if it seems innocuous.

## CASE REPORT

A 70-year-old man visited our hospital with symptoms of painless swelling extending from the buccal mucosa of the submental region to the right angle of the mandible. The bulge first appeared intraorally 4 years ago, gradually growing in size and being accompanied by a thick creamy discharge. The patient started to consume tobacco and smoke cigarettes as a teenager. Besides, he has no insignificant past medical, family history, or dental injuries.

After 4 years of tumour occurrence, the patient was admitted for a thorough examination and evaluation. On clinical examination, a solitary, hard, painless swelling measuring 4 x 4 cm extending from the submental area to the right-sided angle of the mandible was detected above the anterior edentulous alveolar ridge. The buccal vestibule was affected intraorally by its enlargement. The mass was fixed with the underlying structure but free from the overlying structure. There was no tenderness or regional lymphadenopathy. There was no local rise in temperature. The patient has poor oral hygiene and numbness of the lower lip on the right side. On the basis of history and examination, the provisional diagnosis was given as odontogenic tumour and the differential diagnosis was given as odontogenic cyst and bone tumour.

**Figure 1:**
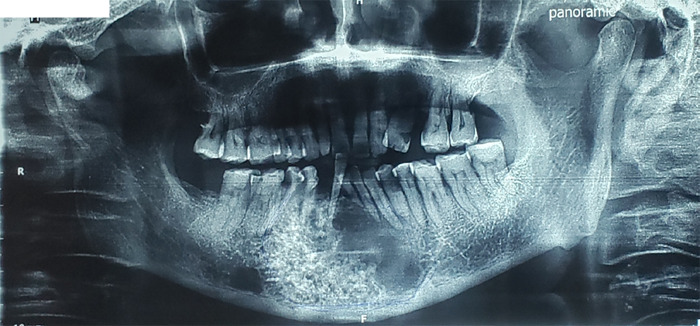
Orthopantomogram showing multilocular radiolucency with a mixed radiographic pattern of honeycomb appearance interspersed with soap bubble pattern in the right mandible.

The patient was explained about the procedure in detail and written informed consent was obtained. The patient was subjected to hematological and radiological examination. Radiographically in orthopantomogram, the lesion showed multilocular radiolucency with a mixed radiographic pattern of honeycomb appearance interspersed with a soap bubble pattern in the right mandible (Figure 1).

**Figure 2:**
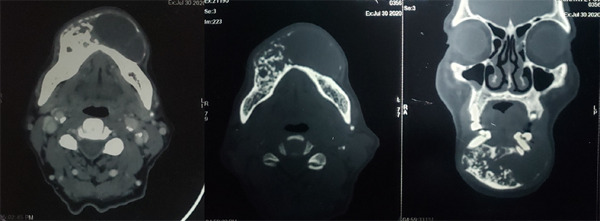
CECT showing an expansile lytic lesion in the mandible region, with enhancing areas noted adjacent to the remaining bone.

**Figure 3:**
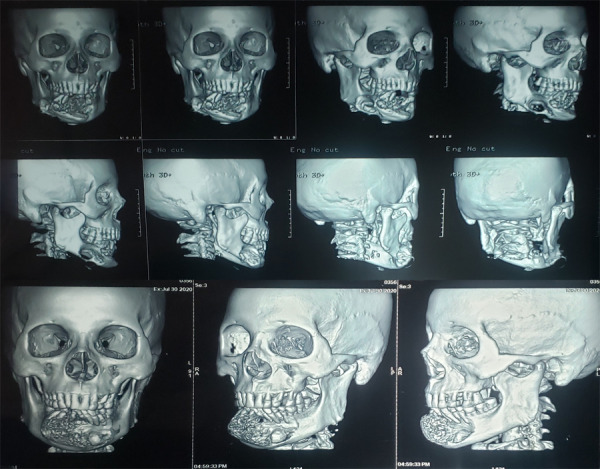
Three-dimensional reconstructed lesion in the mandible.

Contrast-enhanced computed tomography (CECT) scan demonstrated a well-defined expansile mixed density cystic lesion in the body of the mandible measuring 3.6 x 3.9 cm in size. Additionally, mental foramen was not traceable on the right side. Enhancing areas were noted consistent with the solid portion of the tumour and the non-enhancing content inferiorly was consistent with the cystic portion of the tumour (Figure 2 and Figure 3).

Incisional biopsy was taken from the site of the lesion. On hematoxylin and eosin stained soft tissues, cystic lumens lined by ameloblastomatous flattened epithelial lining were found in diverse sites. The epithelium proliferated within the lining as well as into the connective tissue wall, forming ameloblastomatous islands. The cystic lining was formed from the basal layer of the palisading columnar epithelium with reverse polarity. The central portions had stellate reticulum cells, while the rest of the area showed hemorrhage. These features are suggestive of unicystic ameloblastoma of the mandible with bony erosion.

Then the patient was referred to the Department of Oral and Maxillofacial Surgery where he underwent a major local excision with segmental mandibulectomy and flap repair. The bony repair was not done due to the advanced age of the patient. The patient's postoperative course was uneventful, and he was discharged after 14 days. The patient is under strict follow up and no recurrence has been observed so far.

## DISCUSSION

Ameloblastomas are benign odontogenic tumours that are slow-growing, persistent, and locally invasive. They have the ability to grow rapidly, resulting in bone deformities.^1^ Broca published the first detailed description of ameloblastoma in the year 1868. There is no prelidiction for sex. The incidence is highest in the third and fourth decades of life, with the median age of 35.9 years at presentation, and an age range of 4 to 92 years.^4^ The mandible accounts for 80% of instances, the molars and ramus being the most prevalent sites. It presents clinically as a slow-growing, relatively painless expansile tumour that causes tooth mobility, dental displacement, and a deformed facial appearance if left untreated.^1^

Unicystic ameloblastoma is a distinct variant of ameloblastoma that was first described by Robinson and Martinez in 1977. UA refers to those cystic lesions that show clinical and radiologic characteristics of an odontogenic cyst but shows a typical ameloblastomatous epithelium lining part of the cyst cavity, with or without luminal and/or mural tumour proliferation on histological examination.^7^ On histological examination, the UA shows a typical ameloblastomatous epithelium lining part of the cyst cavity, with or without luminal and/or mural tumour growth.^7^ In line with the findings reported by many case reports, the histology of this case also revealed soft tissues, cystic lumens lined by ameloblastomatous flattened epithelial lining which proliferated within the lining as well as into the connective tissue wall, forming ameloblastomatous islands.

The lesion is most commonly observed on the crowns of mandibular third molar teeth, although it can also be present in the interradicular, periapical, and edentulous areas.^8^ In our case, it is associated with the submental area to the right-sided angle of the mandible. It presented as painless swelling with facial asymmetry, dental impaction, tooth displacement, mobility, or tooth resorption.

Radiographically, UA may appear unilocular or multilocular with soap bubble or honeycomb appearance; buccal and lingual expansion of the cortex invariably accompanies ameloblastoma. Thinned and intact cortex shows egg shell appearance.^9^ Similarly, the radiograph of the lesion of this case also identified a well-defined expansile cystic lesion with a mixed radiographic pattern of honeycomb appearance interspersed with soap bubble pattern in the body of the mandible with enhancing thin septa, enhancing soft tissue components along with sclerosis and erosion of symphysis.

Ameloblastoma shows cystic areas of low attenuation and isoattenuating solid regions on computed tomography (CT). CECT scan shows an enhancement effect in the solid components.^10^ In ameloblastoma, there has been a significant correlation between microvessel density (which depicts the vascularization of tumour tissue) and contrast enhancement characteristics in CT scans.^11^ As a result, contrast enhancement is one of the most essential characteristics of benign odontogenic tumours. Because the pattern of bone destruction in intraoral radiographs suggested a tumour rather than a cyst, we decided to use CECT to evaluate the enhancement pattern of the tumour.

Management of ameloblastoma can be done in three ways (1) conservative which includes enucleation and curettage, as well as the use of adjuvant therapies such as Carnoy's solution and cryotherapy, (2) marsupialization, and (3) radical surgery, which includes marginal or block resection (1-1.5 cm margins result in the highest chance of local control) and immediate bone reconstruction. Iliac crest grafts or microvascular fibular flaps may be required for facial reconstruction procedures.^6,12^ Radiotherapy should be considered in patients with positive margins in whom re-resection is not recommended and for those with incompletely resectable tumours. In our case, we performed local excision with segmental resection of the mandible with a safety margin of 1 cm to avoid recurrence and flap repair for reconstruction.

For conventional ameloblastomas, recurrence rates of 50 to 90% have been observed following curettage and up to 15% after marginal or block resection.^3^ For this reason, close and prolonged follow-up examinations should be performed. Conventional radiography and clinical examination may fail to detect recurrences early enough. CT is the most sensitive imaging technique for detecting ameloblastoma, and is to be recommended whenever possible.^10^ Likewise, the recurrence of the lesion in our case was detected by CECT and managed accordingly.

The case highlights the importance of early checkups, imaging, and biopsy for early diagnosis and management of ameloblastoma. Close and prolonged follow-up should be performed for detecting recurrences. Documentation of more such cases makes the pathogenesis of these lesions more insightful. Research is required to correlate the prognosis of these lesions.
